# Lyme Neuroborreliosis: Preliminary Results from an Urban Referral Center Employing Strict CDC Criteria for Case Selection

**DOI:** 10.1155/2010/525206

**Published:** 2010-06-23

**Authors:** David S. Younger, Stuart Orsher

**Affiliations:** ^1^Lyme Neuroborreliosis Research Program, NYU School of Medicine, 333 East 34th Street, Suite 1J, NY 10016, USA; ^2^Department of Medicine, Lenox Hill Hospital, New York, NY 10021, USA

## Abstract

Lyme neuroborreliosis or “neurological Lyme disease” was evidenced in 2 of 23 patients submitted to strict criteria for case selection of the Centers for Disease Control and Prevention employing a two-tier test to detect antibodies to *Borrelia burgdorferi* at a single institution. One patient had symptomatic polyradiculoneuritis, dysautonomia, and serological evidence of early infection; and another had symptomatic small fiber sensory neuropathy, distal polyneuropathy, dysautonomia, and serological evidence of late infection. In the remaining patients symptoms initially ascribed to Lyme disease were probably unrelated to *B. burgdorferi* infection. Our findings suggest early susceptibility and protracted involvement of the nervous system most likely due to the immunological effects of *B. burgdorferi* infection, although the exact mechanisms remain uncertain.

## 1. Introduction

Lyme disease is a multisystem infectious disease caused by the bite of the hard shelled Ixodes tick-borne spirochete *B. burgdorferi* sensu stricto (hereafter referred to as *B. burgdorferi*), which frequently affects the nervous system [1]. Heightened awareness of the spectrum of Lyme neuroborreliosis (LNB) has resulted in increased serological testing to detect antibodies *to B. burgdorferi* in patients with early and late infection, and in many, even without the typical clinical features, who may nonetheless be receiving empiric antibiotics for late disease [2]. With this in mind the first 50 patients with presumed LNB were screened and strict criteria were applied for case ascertainment of Lyme disease. Two patients with varying involvement of the central (CNS), peripheral (PNS), and autonomic nervous system (ANS) and varied duration of LNB emerged. A preliminary report has been published [3]. 

## 2. Methods

The records of 50 consecutive patients referred to the author (D.S.Y.) were selected for inclusion based upon conformity to strict criteria for clinical and laboratory case definition of Lyme disease of the Centers for Disease Control and Prevention (CDC) [4, 5]. The majority of patients were referred with the diagnosis of chronic Lyme disease [6] and had received, or were receiving, antibiotic therapy for persistent Borrelia burgdorferi infection. Altogether, 23 patients underwent two-tier serological testing for the diagnosis of Lyme disease at the single reference laboratory, Stony Brook University Laboratory, New York, employing a first-tier screening enzyme-linked immunoassay (ELISA) and second-tier IgG and IgM immunoblots performed on reactive or borderline results as recommended by the CDC [5]. The 27 patients that had laboratory studies for Lyme disease performed elsewhere were excluded from this analysis. Of the remaining 21 patients, 5 with nonreactive first-tier ELISA as well as 12 with reactive and 4 with borderline reactive screening serology, all with nonconfirmatory Lyme IgG or IgM immunoblots, were also excluded. Two patients described below met criteria for case selection. 

Noncontrast magnetic resonance imaging (MRI) and nuclear medicine (NM) cerebral perfusion imaging with single-photon emission spectroscopy (SPECT) screened for brain dysfunction, the main symptoms of which were typically neurocognitive. 

Quantitative sensory testing (QST) for heat pain perception thresholds [7] and epidermal nerve fiber (ENF) studies of the thigh and calf [8] screened for small fiber sensory nerve (SFSN) dysfunction [9], the main symptom of which was dysesthesia, often reported as tingling, prickling, burning, deep aching, jabbing, or shooting sensations often in association with numbness and coldness of the limbs. 

Quantitative sudomotor axon reflex testing, beat-to-beat blood pressure (BP), and heart rate responses to head-up tilt, deep breathing, Valsalva maneuver, with calculation of a composite autonomic scoring scale (CASS) using a WR Electronics laboratory, Rochester, Minnesota, [10] screened for ANS dysfunction, the symptoms of which consisted of postural hypotension, palpitation, dizziness, headache, and lightheadedness. 

Electrodiagnostic studies including nerve conductions and electromyography of the arms and legs defined PNS dysfunction including distal polyneuropathy (DPN) [11] and polyradiculoneuritis [12] in the two patients, the symptoms of which included patchy radicular peripheral nerve disturbances often with little or no motor involvement. 

Cerebrospinal fluid (CSF) was not collected for diagnostic levels of IgM and IgG antibodies alone or in paired analysis with serum, or for isolation of B. burgdorferi using polymerase chain reaction studies, in either patient, and thus was not available for retrospective analysis. 

## 3. Patient Descriptions

### 3.1. Patient 1

A previously normal 38 year old woman had lightheadedness, dizziness, palpitation, and headache commencing 3 months after tick bite, fever, joint pain, and erythema migrans (EM) rash in the summer of 2007 that prompted the clinical diagnosis of Lyme disease. Neurological examination showed slight tandem imbalance, stocking cold temperature, and vibratory sensory loss with otherwise normal cognition, cranial nerves, limb strength, coordination, and reflexes. Laboratory testing showed reactive Lyme serology with an optical density (OD) of  .263 (reactive cutoff + 3 standard deviations (SD)  .130), positive IgM immunoblot comprised of 23 and 41 kDa bands, and a negative IgG immunoblot. Noncontrast brain MRI showed a few punctuate scattered supratentorial white matter signal abnormalities. Cerebral perfusion with SPECT showed mildly diminished blood flow in the posterior temporal and parietal lobes. Electrodiagnostic studies of the left arm and legs showed patchy polyradiculoneuritis. Epidermal nerve fiber studies and QST of the left hand and foot were normal. Autonomic studies showed exaggerated postural tachycardia on active stand and tilting for 10 minutes, with abnormal fall in BP during phase II of the Valsalva maneuver with an overall CASS of 1. She was treated with oral doxycycline 100 mg bid for one month with steady mild subjective improvement that was sustained over the ensuing three months although repeat electrodiagnostic and autonomic neurophysiological testing were not performed.

### 3.2. Patient 2

 A previously normal 48 year old woman was diagnosed with Lyme disease following a tick bite and EM rash and treated with oral followed by intravenous antibiotics according to prevailing standards [13]. One year later she noted cognitive impairment, imbalance, and sensory disturbance in the legs. Lyme serological studies were reactive with an OD of 1.201 (reactive cutoff  .149), positive Lyme IgG immunoblot comprised of 18, 28, 30, 41, 45, 58, and 93 kDa bands, and negative IgM immunoblot. Neurological examination showed mild stocking vibratory and cold temperature sensory loss with otherwise normal cognition, cranial nerves, limb strength, coordination, and reflexes. Electrodiagnostic studies including EMG/NCS showed a distal sensorimotor neuropathy with mixed demyelinating and axonal features. Neuropsychological studies revealed significant deficits in semantic fluency, reading speed and comprehension, auditory attention, visual and verbal memory, psychomotor speed, and phonemic verbal fluency, without depression or anxiety. Noncontrast brain MRI was normal. Quantitative sensory testing showed heat pain thresholds below the 5th percentile in the left foot and normal in the left hand. Cerebral perfusion with SPECT showed decreased perfusion in the temporal lobes. Autonomic testing showed mild phase IV attenuation of the Valsalva maneuver with an overall CASS of 1. She was not treated with further antibiotics. Sustained subjective and objective neurological improvement on repeat examination 3 months later paralleled improvement on repeat autonomic neurophysiological studies and QST after an empiric course of 2 grams per kilogram of intravenous immunoglobulin (IVIg) in 5 successive days per month for 3 months for acquired autoimmune peripheral and autonomic neuropathy.

## 4. Discussion

Two patients met CDC criteria for the clinical and laboratory diagnosis of Lyme disease, including one with symptomatic polyradiculoneuritis, dysautonomia, and serological evidence of early infection and the other with SFSN, DPN, dysautonomia, and serological evidence of late infection. The remaining 21 patients had negative or indeterminate laboratory evidence of Lyme disease indicating that symptoms initially ascribed to Lyme disease were probably unrelated to *B. burgdorferi* infection. The CDC [4, 5] has not determined the sensitivity or specifity of the serological diagnosis of Lyme disease in any given cohort, using diagnostic levels of IgM and IgG antibodies to the *B. burgdorferi* spirochete in serum. Thus, it is uncertain whether having 2 of 23 patients (8.7%) with diagnostic serology for Lyme disease in a given cohort with primarily chronic symptoms is representative of our cohort. A two-tier test approach for active disease and previous infection with the demonstration of a significant change in IgM or IgG antibody response to *B. burgdorferi* in paired acute- and convalescent-phase serum samples, examination of diagnostic levels of IgM and IgG antibodies to the spirochete in CSF, and isolation of *B. burgdorferi* from CSF are recommended to improve the diagnostic accuracy of serological testing in Lyme disease [4, 5], including LNB. 

Unlike meningitis, cranial neuritis, polyradiculoneuritis [14], and encephalomyelitis [15] which have been ascribed to the direct effects of *B. burgdorferi* infection, the etiopathogenesis of encephalopathy, SFSN, DPN, and dysautonomia are less certain, and probably related to autoimmune factors, triggered by exposure to *B. burgdorferi* antigens. Two candidate antigens that cross-react with constituent peripheral nerve molecules, one against flagellin and the other against gangliosides experimentally, are found in the sera and peripheral nerve of affected patients [16–18]. Acquired autonomic neuropathy, in which autonomic fibers are selectively or disproportionately affected, is presumably also of autoimmune cause as suggested by the occurrence of autonomic neuropathy after Lymerix and the Connaught vaccination [19, 20], and the favorable response to IVIg therapy in Patient 2. 

The management of LNB remains controversial as to the timing and duration of oral and intravenous antibiotics. The occurrence of peripheral neuropathy, dysautonomia, and encephalopathy years later after adequate antibiotic therapy underscores the selective vulnerability of the nervous system to the immunological effects of *B. burgdorferi* infection, although the exact mechanisms remain uncertain. 
